# Exploring the longitudinal impacts of academic stress and lifestyle factors among Chinese students

**DOI:** 10.1080/20523211.2024.2398706

**Published:** 2024-09-06

**Authors:** Wang Han, Ali Altalbe, Nadia Rehman, Shazia Rehman, Samantha Sharma

**Affiliations:** a School of the Community for Chinese Nation, North Minzu University, Ningxia, China; b Department of Computer Engineering, Prince Sattam bin Abdulaziz University, Al-Kharj, Saudi Arabia; c Faculty of Computing and Information Technology, King Abdulaziz University, Jeddah, Saudi Arabia; d Department of Mathematics, COMSATS University, Islamabad, Pakistan; e Department of Psychiatry, National Clinical Research Center for Mental Disorders, and National Center for Mental Disorders, The Second Xiangya Hospital of Central South University, Changsha, People’s Republic of China; f Hunan Key Laboratory of Psychiatry and Mental Health, Hunan Medical Center for Mental Health, China National Technology Institute on Mental Disorders, Hunan Technology Institute of Psychiatry, Mental Health Institute of Central South University, Changsha, People’s Republic of China; g Department of Psychology, Wuhan University, Wuhan, People’s Republic of China

**Keywords:** Academic stress, bidirectional association, longitudinal relationship, mental health history, physical activity, time management skills

## Abstract

**Background:**

Several cross-sectional and longitudinal investigations have demonstrated a robust association between academic stress, physical activity, mental health history, and time management skills. However, the existing literature exhibits inconsistencies in the relationship between academic stress and its predictive effects on physical activity and mental health history. In addition, there is a scarcity of scholarly research that concentrates on the significance of time management skills within this particular context. Furthermore, limited research has investigated these variables’ longitudinal associations and causal pathways. Therefore, the present research explores the longitudinal relationships among academic stress, physical activity, mental health history, and time management skills among university students.

**Methods:**

The data were gathered from Wuhan University, China, employing a two-wave longitudinal survey methodology with an annual interval. A cohort of 980 university-level students engaged in the completion of questionnaires, which encompassed measures of academic stress via the Educational Stress Scale for Adolescents (ESSA), physical activity ascertained through Cho's five-item questionnaire, mental health history assessed by the Kessler Psychological Distress Scale, and time management skills evaluated using the Time Management Behaviour Scale (TMBS). Subsequently, a cross-lagged path model was utilised to examine the prospective associations among these constructs.

**Results:**

The outcomes of the cross-lagged path analysis indicated the presence of significant bidirectional relationships between academic stress and physical activity, mental health history, and time management skills. In addition, bidirectional interconnections existed between physical activity and mental health history. Furthermore, unilateral correlations were detected between physical activity and time management skills.

**Conclusions:**

These findings underscore the importance of an integrated approach to student health initiatives and highlight the need for comprehensive support systems that address student well-being's psychological and physical aspects.

## Introduction

Stress is a prevalent concern that affects individuals at various points. One prevalent form of stress experienced by university students is academic stress (Bedewy & Gabriel, 2015). A significant potential exists for detriment to a university student's academic achievement. The existing literature has highlighted that stress can be attributed to timely assignment submission, GPA values, modular grades, and loss of hopes and ambitions (Barker et al., 2018; Hj Ramli et al., 2018). Likewise, the research has explored the coping strategies utilized by university students to handle academic stress effectively. The identified strategies include engaging in activities such as listening to music, watching videos, maintaining motivation, diligently working, and employing wishful positive thoughts (Hamdan-Mansour et al., 2009; Misra et al., 2000; Saklofske et al., 2012). Positive mental health may not have received much research attention; however, it is significant in understanding the overall mental well-being of any individual (Margraf et al., 2020; Tennant et al., 2007). To provide additional substantiation for this concept, Margraf et al. (2020) have demonstrated that robust positive mental health significantly predicts both adverse and beneficial mental health outcomes over an extended period. Investigating the relationship between academic stress and mental well-being is crucial, as previous research has demonstrated that poor mental well-being has a detrimental effect on college academic performance. Therefore, further exploration of this correlation is essential (Córdova Olivera et al., 2023; Ma et al., 2024; Wu et al., 2020). Examining academic stress by researchers allows for identifying factors that may increase risk and those that may provide protection and implementing coping strategies (Rehman et al., 2024). This enables the establishment of evidence-based treatments to improve students’ psychological well-being and academic improvements. Besides, it is critical to understand the impacts of academic stress on students to foster a culture of mental health awareness and promote it within the educational center. Implementing a preventative approach can minimize the deterioration of mental health concerns and provide a pleasant academic environment for students.

Several hypotheses suggest that physical activity improves academic achievement, such as arousal and attentiveness. This implies that when you exercise or are involved in any physical activity, your body becomes more aroused, producing more neurotransmitters that aid in focusing and paying concentration. This includes dopamine, which is associated with attention and focus (Lambourne & Tomporowski, 2010; Tomporowski, 2003). Another theoretical perspective posits that physical exercise could enhance cognitive functioning by promoting simultaneous engagement in cognitive and motor tasks, facilitating improved focus and attention. It is plausible that engaging in physical activity may contribute to enhancements in one's executive function (Budde et al., 2008). Evidence from the literature demonstrates an association between physical activity and improved cognitive functioning, encompassing attention, memory, and information processing. An overwhelming body of literature suggests that students involved in sufficient regular physical activities exhibit improved levels of academic performance, including improved grades and standardised test scores (Hillman et al., 2008; Irwin, 2004; Monserrat-Hernández et al., 2023; Singh et al., 2012). These behavioural effects are particularly apparent in educational contexts, where learning and academic success depend heavily on sustained attention, efficient information processing, and memory retrieval. Investigations on the connections between educational performance/achievements and physical activity substantially influence how educational initiatives are established (Álvarez-Bueno et al., 2017; Monserrat-Hernández et al., 2023). Throughout the school day, physical activities, such as physical, educational programmes, energetic breaks, or extracurricular athletics, can be a blended approach to improve students’ cognitive functioning and, ultimately, educational performance overall (Rasberry et al., 2011; Teuber et al., 2024). Many theoretical frameworks and empirical evidence indicate a considerable relationship between academic success and physical activities (Kizlik et al., 2018; Wahid et al., 2023). Policy-makers and academics may wish to investigate integrating evidence-based physical activity programmes to boost educational environments and foster the holistic well-being of students.

Time management refers to the capacity to allocate and organise time effectively to accomplish tasks within specified deadlines (Mancini & Mancini, 2003). This concept is essentially synonymous with self-management. The competencies necessary for effectively overseeing others are also essential for self-management, characterised by the capacity to strategize, mobilise resources, provide clear direction, and exercise authority (Rani & Sharma, 2018). The acquisition of this skill is often overlooked despite its potential for instruction. How students perceive and manage time can yield favourable and unfavourable repercussions on their personal and academic achievements (MUHAMMAD JEHANGIR Alyami et al., 2021; Khan et al., 2020). A cross-sectional research study examined the relationship between time management skills and academic performance. The findings indicated that training time management skills significantly impact academic performance and self-efficacy (Sevari & Kandy, 2011). The evidence from existing literature has reported a significant negative correlation between time management skills and academic stress levels among university students, signifying that students who exhibit practical time management skills tend to experience reduced stress levels associated with their academic pursuits (Manjula, 2016; Sallehuddin et al., 2019). Likewise, in a non-equivalent dependent variable design, the researcher demonstrated that students who underwent training in time management techniques exhibited considerable enhancements in their capacity to manage academic workloads and reported decreased academic stress levels (Häfner et al., 2015). These facts demonstrate how important it is to incorporate the development of time management skills into the curriculum to provide students with the tools they need to handle academic pressures successfully. Efficient time management entails the recognition of the value of time, the establishment and ranking of both long-term and short-term objectives, and the continual monitoring of one's advancement. Research has demonstrated that this approach enhances academic achievement, boosts overall satisfaction, and reduces stress and emotional fatigue (Gayef et al., 2017; Karakose, 2015).

### The present study

In cross-sectional studies, the extant literature has investigated the distinct influences of physical activity, mental health history, and time management skills on academic stress. However, there exists a research gap in the longitudinal examination of the cumulative impacts of these factors among university students in China. This research represents the first of its kind, exploring the directional interconnections among academic stress, physical activity, mental health history, and time management skills within a Chinese university student population over one year while controlling for age and sex. This study aims to elucidate the longitudinal relationships to offer valuable insights for developing targeted interventions to support students’ well-being and academic success.

## Methods

### Study design and participants

To carry out a questionnaire-based survey, the study employed a stratified cluster sampling approach to select a representative sample of university students across various majors and disciplines at Wuhan University in Wuhan, China. Therefore, we initiated communication with the university administration and, with their assistance, acquired a comprehensive roster of all available classes. Subsequently, we utilised a random selection process to choose ten classes from various academic years.

We employed a cluster sampling technique for its pragmatism and efficacy, enabling data aggregation across two-time points spaced one year apart. Cluster sampling allowed us to streamline data collection procedures while encompassing a comprehensive range of academic fields and student demographic variables. Through the randomised selection of clusters, we endeavoured to mitigate sampling bias, thereby bolstering the dependability and validity of our results. This strategy ensures that our sample remains representative and that the outcomes can be extrapolated to analogous university contexts.

To be eligible for inclusion in the study, participants were required to be at least 18 years of age, currently enrolled as university students, report no familial history of mental illness (anxiety or depression), and not have received a clinical diagnosis for addictive, substance-abusive, or affective disorders (self-reported).

The initial phase of the survey *(P**_1_**)* was conducted in September 2022, with 1,012 student participants. Subsequently, a second data collection phase *(P**_2_**)* took place one year later, in which 980 students completed the questionnaire again. However, 32 participants were excluded from the analysis due to absence from class during the data collection period. The surveys were administered in a classroom setting, with trained investigators facilitating the completion of written questionnaires by participants within 30 minutes. The guidance provided by the investigators did not include deliberation on the questionnaire's content but instead focused on instructions for completion, such as indicating where to record responses and timing the test takers.

### Ethical consent

The study was granted ethical approval from the Institutional Review Board (IRB) of Wuhan University, China, and was conducted according to the guidelines and regulations of the Declaration of Helsinki and its later amendments. The participants provided written informed consent to participate in this study before completing the questionnaire.

## Measures

### Sociodemographic

The survey administered a sociodemographic questionnaire to gather information on participants’ age, gender, community type (urban or rural), and field of discipline (science and engineering, medicine, or social science).

### Academic stress

The survey adopted the Educational Stress Scale for Adolescents (ESSA) developed by Sun et al. (2011) to assess the level of academic stress perceived by the study participants. The survey questionnaire consists of 16 statements assessed using a 5-point Likert scale, with responses 1: strongly disagree to 5: strongly agree. Sample items include ‘I feel pressured by the academic expectations of my teachers' and ‘I worry about my academic performance.' The composite score demonstrates considerable variability within the range of 16–80, with higher scores indicative of elevated levels of perceived stress. This scale comprises distinct dimensions, including pressure related to academic study, workload, concern about grades, stress from self-imposed expectations, and feelings of despondency. The ESSA has been validated in various populations, including Chinese students, ensuring its appropriateness for this study (Sun, 2012; Sun et al., 2013; X. Zhu, Haegele, et al., 2021).

### Physical activity

Participants’ physical Activity based on type, intensity, duration, frequency, and total length was evaluated through Cho’s five items physical activity questionnaire, which is a valid and reliable scale as reported in prior research (Aghababa et al., 2021; Cho, 2014; Cho, 2016; Kayani et al., 2021). The responses were recorded on a 5-point Likert scale. An initial score was derived by aggregating the responses related to the overall duration, frequency, intensity, and length of physical activity to estimate the level of physical activity. The combined scores were subsequently multiplied by the standardised score corresponding to the student's particular type of physical activity. The range of scores observed was 4–100. Physical activity levels were classified as follows:

- Very high > 96, high (64–95), acceptable (36–63), low activity (16–35), and inactive (4–15).

### Mental health history

The Kessler Psychological Distress Scale – K10 (Kessler et al., 2002) was adopted in the current survey to evaluate the mental health history of the study participants, which has already been validated in the Chinese population (Ren et al., 2021; G. Zhu, Wang, et al., 2021; M. Zhang et al., 2018; J. Chen, 2012). The instrument consists of ten items intended to evaluate manifestations of depression and anxiety experienced over the last 30 days. The sample items include ‘In the past 30 days, how often did you feel tired out for no good reason?' and ‘In the past 30 days, how often did you feel nervous?' The study’s participants were instructed to rate the frequency of their experience on a 5-point Likert scale, with response options 1: never to 5: always.

### Time management behavior scale (TMBS)

This study utilized the four-factor scale developed by Macan et al. (1990), a widely employed scale by researchers worldwide to evaluate students’ perspectives on time management behavior. The scale consists of four factors related to goal-setting and prioritization: scheduling and planning mechanics, organizational preference, and perceived time control. Participants were instructed to rate their responses on a Five-Point Likert scale, with options ‘1: seldom true' to ‘5: very often true'. Sample items include statements such as ‘I set specific goals before I begin a task' and ‘I keep a daily log of activities’.

## Statistical analysis

The initial analysis phase involved evaluating the data distribution via skewness and kurtosis. This was followed by descriptive analysis and bivariate correlational assessment using SPSS (v27). Before executing cross-lagged panel analysis, an evaluation was undertaken to ascertain the longitudinal measurement invariance of the studied constructs. Subsequently, a cross-lagged model analysis was utilized to explore the longitudinal bidirectional associations between academic stress, physical activity, mental health history, and time management skills while controlling for age and gender effects.

The adequacy of model fit was evaluated through the comparative fit index (CFI), Tucker – Lewis index (TLI), root – mean – square error of approximation (RMSEA), and standard root – mean – square (SRMR). Additionally, 95% confidence intervals (CI) were computed using a bias-corrected bootstrap sample that underwent 5000 iterations. The 95% CI did not include zero, indicating an observed statistically significant effect at *p* < 0.05. Furthermore, the change values of CFI (ΔCFI) and RMSEA (ΔRMSEA) were employed as measures for assessing the measurement invariance. When the ΔCFI is less than or equal to 0.01, and the ΔRMSEA is less than or equal to 0.015, the measurement invariance model was deemed to be acceptable (F. F. Chen, 2007).

## Results

Table 1 displays the descriptive analysis of the chosen study variables. The statistical analysis demonstrated normality in the variables, as indicated by the skewness values remaining below ±2 and the kurtosis values remaining below ±7.

**Table 1.** Descriptive analysis of the study variables.

	Mean	SD	Skewness	kurtosis
Academic stress P1	2.54	1.34	1.22	−1.76
Physical activity P1	2.01	0.71	−0.87	−2.40
Mental health history P1	2.35	0.87	1.37	−3.04
Time management skills P1	24.92	3.28	−1.95	−0.95
Academic stress P2	2.33	0.98	1.65	−2.17
Physical activity P2	2.15	0.93	−0.74	−0.89
Mental health history P2	2.53	1.17	1.25	−2.46
Time management skills P2	25.16	2.28	−1.63	−2.09

Note: P1 denotes the baseline period, and P2 denotes the second period. SD: standard deviation.

Table 2 presents the findings of the reliability analysis, along with an evaluation of the discriminant and divergent validity of the study variables. The reliability of the analysis was assessed by employing Cronbach's alpha coefficient and McDonald's omega. The evaluation of construct validity entailed the analysis of model fit statistics and the average variance extracted index. The research findings indicated satisfactory reliability and validity for the ESSA, PA, TMBS, and K10 constructs at two different time points, i.e. *P_1_* and *P_2_*.

**Table 2.** Discriminant validity, reliability, and construct validity analysis results

	ESSA	PA	TMBS	K10	Cut off
**At baseline P1**					
Basement effect	3.5%	2.7%	1.8%	1.5%	<15%
Ceiling effect	2.9%	3.4%	4.6%	2.5%	<15%
McDonald’s omega	0.81	0.82	0.82	0.86	≥0.7
Cronbach’s alpha	0.89	0.88	0.88	0.90	≥0.7
Ferguson’s delta	0.93	0.95	0.94	0.94	≥0.9
χ2/df	1.43	1.67	2.52	2.16	<3
CFI	0.99	0.97	0.97	0.96	≥0.95
TLI	0.95	0.94	0.96	0.94	≥0.95
RMSEA	0.05	0.05	0.06	0.05	<0.08
SRMR	0.04	0.04	0.04	0.04	<0.08
CR	0.93	0.94	0.93	0.95	≥0.7
AVE	0.69	0.70	0.59	0.56	>0.5
**At the second time P2**					
Basement effect	2.9%	3.1%	2.1%	1.9%	<15%
Ceiling effect	3.6%	2.8%	3.8%	2.7%	<15%
McDonald’s omega	0.83	0.84	0.84	0.87	≥0.7
Cronbach’s alpha	0.92	0.90	0.91	0.93	≥0.7
Ferguson’s delta	0.94	0.95	0.96	0.95	≥0.9
χ2/df	1.65	1.78	2.46	2.32	<3
CFI	0.99	0.98	0.98	0.99	≥0.95
TLI	0.96	0.96	0.95	0.97	≥0.95
RMSEA	0.05	0.06	0.05	0.05	<0.08
SRMR	0.04	0.04	0.03	0.04	<0.08
CR	0.95	0.92	0.93	0.98	≥0.7
AVE	0.56	0.67	0.62	0.59	>0.5

Note: CFI: Comparative fit index, TLI: Tucker – Lewis index, RMSEA: Root – mean – square error of approximation, SRMR: Standard root – mean – square, CR: Construct reliability, AVE: Average variance extracted.

Table 3 demonstrates the bivariate relationship among study variables across two time frames. Academic stress and physical activity exhibited a strong negative relationship at both periods, suggesting that higher physical activity levels were associated with lower academic stress levels. Across both time points, there was a consistent negative correlation between mental health history and physical activity and time management skills, whereas a significant positive association between academic stress and mental health history of study participants. This suggests that a history of mental health issues may be associated with increased or decreased physical activity and ineffective time management. Moreover, the results showed a significant inverse relationship between academic stress and time management skills, suggesting that higher time management skills are linked to lower stress levels. Over the two time periods, the correlation patterns were largely steady, suggesting that these variables had stable associations over time.

**Table 3.** Bivariate correlation analysis results.

		1	2	3	4	5	6	7	8
1	Academic stress P1	–							
2	Physical activity P1	−0.412**	–						
3	Mental health history P1	0.322*	−0.404**	–					
4	Time management skills P1	−0.465**	−0.247*	−0.209*	–				
5	Academic stress P2	0.604**	−0.319**	0.339*	−0.425**	–			
6	Physical activity P2	−0.376**	−0.559**	−0.532**	−0.142	−0.422**	–		
7	Mental health history P2	0.392**	−0.328*	−0.436**	−0.449**	0.495**	−0.572**	–	
8	Time management skills P2	−0.415**	−0.244*	−0.451**	−0.563**	−0.557**	−0.258*	−0.255*	–

Note: P1 denotes the baseline period, and P2 denotes the second period. **p < 0.001. *p < 0.01.

**Figure 1.**
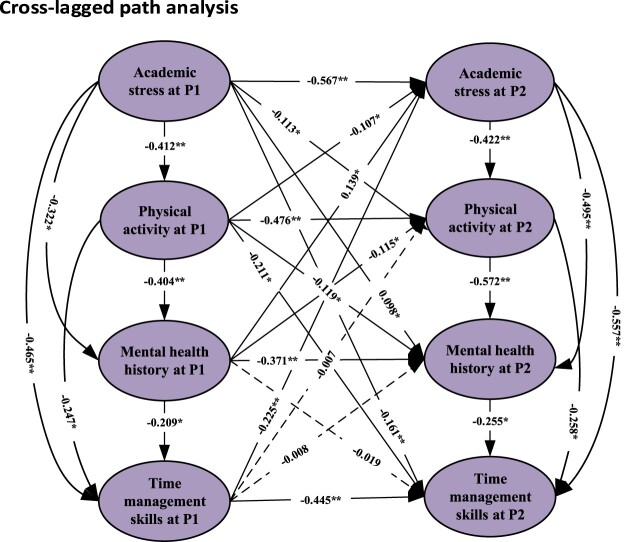
Cross-lagged path analysis of academic stress, physical activity, mental health history, and time management skills of study participants across two time frames (P1: baseline time frame and P2: second time frame). The dotted line shows insignificant paths; straight line shows significant paths. **p < 0.001. *p < 0.01.

Significant bidirectional associations from academic stress to physical activity, mental health history, and time management skills were observed using the cross-lagged path analysis over two time frames, suggesting that these factors predict one another over time. Physical activity was first found to be inversely associated with time management skills, but this association did not reverse, indicating a one-way relationship between physical activity and time management skills. This research limited the effects of mental health history, suggesting that it may have a less direct or meaningful effect than other variables (Figure 1).

## Discussion

We employed a two-wave longitudinal approach in the present investigation and established a cross-lagged framework to investigate the reciprocal relationships between time management abilities, physical activity, mental health history, and academic stress. The results of this research contribute to a deeper understanding of the longitudinal relationships and directional effects among these variables. The outcomes demonstrate longitudinal associations in both directions, i.e. (a) academic stress with physical activity, mental health history, and time management skills, and (b) physical activity with mental health history. To the best of the author's knowledge, the present research represents the novel investigation in which academic stress exhibited bidirectional relationships with physical activity, mental health history, and time management skills. Additionally, unilateral associations were observed between physical activity and time management skills.

The outcomes demonstrated a bidirectional relationship between academic stress and participant’s physical activity, mental health, and time management skills, corroborated by several works in the literature. Research has proven that physical activity enhances psychological health and lowers perceived stress levels, possibly due to endorphin production. Also, the notion of self-regulation argues that efficient self-regulation methods may minimise responses to stress, which explains the association between improved time management abilities and less academic stress. Furthermore, previous experience with psychological issues has been associated with a higher susceptibility to stress, supporting the diathesis-stress hypothesis that suggests a person's history of vulnerability might influence how they react to stressors (Broekman, 2011; Cheng et al., 2016). Burns et al. (2018) highlight recognising the interconnected link between stress and physical activity. Increased academic stress can lead to reduced activity levels, while inadequate physical activity can worsen stress. This two-way connection underscores the necessity of initiatives that promote activity among college students and assist them in coping with pressure. Teuber et al. (2024) revealed fluctuations in stress levels and physical activity patterns over time. It was found that students tended to engage less in activities on days when they reported feeling more stressed about their academic performance. This highlights how college students might turn to exercise to manage the stress linked to their responsibilities. The researchers also witnessed several vital aspects of academic stress and university students’ physical activity. The investigators observed themes such as time management, the perceived advantages of exercising, and academic achievement. Almost all available literature has shown that motivating physical activity among learners would be an excellent way to reduce academic stress. By acknowledging this fact, institutions can improve their capacity to address both scholastic performance and the general healthiness of their students concurrently to prevent such outcomes from occurring again.

Numerous studies have investigated the correlation between mental health and academic pressure. Shen (2020) posits a relationship between escalated stress levels and a corresponding rise in anxiety and depressive symptomatology among students, underscoring the interdependent association between academic stress and physiological wellness. Conversely, it has been observed that compromised mental health can intensify stress, engendering a cyclical pattern that impinges upon holistic well-being and scholastic achievement. Zhang et al. (2022) corroborated the positive correlation between academic stress and students’ psychological welfare, furnishing empirical substantiation. Analogously, Liu et al. (2022) underscored the imperative of recognising the enduring consequences of the reciprocal interaction between academic stress and subsequent mental health complications. This elucidates the importance of timely interventions and sustained support in mitigating the progression of academic stress and mental health challenges within university settings.

Our findings demonstrated a significant bidirectional association between academic stress and time management skills in our sample, emphasising a significant area of the subject with considerable consequences for students’ wellness and academic achievement. The work of Rani and Sharma (2018) aligns with our research outcomes, highlighting the importance of efficient time management in mitigating academic stress among student demographics. According to their findings, students who employ efficient time management practices experience reduced stress levels related to academic workloads and deadlines. Additionally, findings from other observational studies corroborate the conclusions of our research, indicating that effective time management strategies yielded a significant reduction in academic stress when compared to engagement in leisure pursuits (Dong et al., 2024; Häfner et al., 2015; Macan et al., 1990; Mussarat Jabeen Khan et al., 2013). Demonstrating an individual's time management disposition may indicate their psychological and behavioural traits related to their time utilisation. The capacity to proficiently monitor and regulate one's time facilitates individuals judiciously prioritising tasks according to their goals, allocating substantial time to critical learning endeavors, and exhibiting adept self-discipline. Assessing one's perception of time efficacy can aid individuals in evaluating their personal time management abilities. When individuals demonstrate skillful time management and achievement of their goals, it is probable that they will encounter positive emotions, heightened overall well-being, and improved academic performance (Dong et al., 2024).

Moreover, the present study observed a unilateral association between physical activity and the mental health history of participants, which corroborates several studies in the extant literature emphasising the salubrious effects of physical activities on various mental health outcomes, including stress, anxiety, and depression (Al-Hazzaa & AlMarzooqi, 2018; Holmes et al., 2016; Jones et al., 2017; Mahindru et al., 2023; Sumińska, 2021). Drawing from these statistics, it becomes evident that individuals who engage regularly in physical activity exhibit a lower prevalence of mental health issues compared to their counterparts with limited physical activity. The prevailing body of research furnishes compelling evidence substantiating the efficacy of physical activity in diminishing energy levels, fostering the discharge of frustration, and alleviating muscle tension (Holmes et al., 2016; Mahindru et al., 2023; Stubbs et al., 2017). Engaging in physical activity amplifies endorphin levels, often called the ‘happiness hormone,' and elevates cortisol and norepinephrine levels, typically associated with stress and anxiety (Rebar et al., 2015). Further, scholarly investigations indicate that participation in physical activity is instrumental in mitigating depressive symptoms and modulating intricate emotional processes (McMahon et al., 2017; Rehman et al., 2024). The findings underscore the potential of physical activity engagement as a prophylactic measure against adverse mental health outcomes and underline the importance of integrating physical activity into interventions designed to address mental health concerns.

### Practical implications

The longitudinal associations elucidated in our research between academic stress, physical activity, mental health history, and time management skills among university students offer profound implications for intervention strategies. These findings underscore the potential efficacy of initiatives to mitigate academic stress through elevated awareness. Universities could pioneer campaigns advocating increased physical activity and reinforcing robust time management acumen. With this insight, students can make informed decisions that foster optimal time utilisation and active participation in wellness-promoting activities.

Furthermore, proactive measures to address the root causes of academic stress can be ameliorated by conducting screenings for mental health histories coupled with the provision of targeted care. Integrating holistic wellness programmes that converge academic support, emotional counseling, and physical health services can substantially enhance students’ academic achievement and comprehensive well-being. Such multifaceted approaches, grounded in empirical evidence, promise to reshape the educational landscape by prioritising student wellness and resilience.

### Limitations

Although the results of this investigation show longitudinal relationships between the variables under investigation, several limitations must be considered while interpreting the results. Initially, the results can only be partially generalised since the sample, limited to students from a particular university, might not accurately reflect the larger population of university students. Secondly, response biases may be introduced, compromising the validity of the established relationships when self-reported information is used to measure variables like stress levels and physical activity. Third, despite finding significant correlations among the selected variables in our study, it is essential to emphasise that these findings are based on correlational data. As a result, the determination of causation continues to be elusive and remains inconclusively ascertained. Furthermore, the longitudinal approach makes drawing an unambiguous conclusion on causality challenging while providing insightful information about how relationships between variables change over time. If the characteristics of individuals who finished both assessments differed from those who did not, turnover across the research period might create bias.

## Conclusion

Based on the observed longitudinal relationships between academic stress, physical activity, mental health history, and time management skills among university students, the present research emphasises the interdependence of these variables in affecting student wellness and academic achievement. The bidirectional associations discovered in our research demonstrate that efforts to lower academic stress should include initiatives to promote physical activity, address mental health challenges, and strengthen time management skills in addition to stress management approaches. These statistics highlight the significance of an integrated framework for student health initiatives and the demand for all-encompassing support networks that address student life's psychological and physical facets.

## Data Availability

The data are available from the corresponding author upon reasonable request.
